# Comparison of dorsal closing wedge calcaneal osteotomy versus posterosuperior prominence resection for the treatment of Haglund syndrome

**DOI:** 10.1186/s13018-020-01687-6

**Published:** 2020-05-07

**Authors:** Zilu Ge, Lin Ma, Hong Tang, Mingyu Yang, Aining Yang, Chengsong Yuan, Xu Tao, Binghua Zhou, Kanglai Tang, Wan Chen

**Affiliations:** Department of Orthopaedics/Sports Medicine Center, State Key Laboratory of Trauma, Burn and Combined Injury, Southwest Hospital, Third Military Medical University (Army Medical University), Gaotanyan Street 30, Shapingba District, Chongqing, 400038 China

**Keywords:** Haglund syndrome, Dorsal closing wedge calcaneal osteotomy, Posterosuperior prominence resection

## Abstract

**Background:**

Haglund syndrome is a common disease that causes posterior heel pain. This study compared the clinical outcomes of dorsal closing wedge calcaneal osteotomy (DCWCO) and posterosuperior prominence resection (PPR) for the treatment of Haglund syndrome.

**Methods:**

This retrospective study included 12 patients who underwent DCWCO and 32 patients who underwent PPR from January 2010 to August 2016. Patients were evaluated using the American Orthopedic Foot Ankle Society ankle-hindfoot scale (AOFAS), Victorian Institute of Sport Assessment Scale for Achilles tendinopathy (VISA-A), Fowler-Philip angle, Bohler’s angle, and calcaneal pitch angle preoperatively and postoperatively (at 3 months, 6 months, 1 year, and the latest follow-up).

**Results:**

Both groups exhibited a significant increase in their AOFAS and VISA-A scores after surgery. The DCWCO group had lower AOFAS scores than the PPR group at 6 months (77.6 ± 5.1 vs. 82.8 ± 7.8; *P* = 0.037) but had higher scores at the latest follow-up (98.2 ± 2.3 vs. 93.4 ± 6.1; *P* = 0.030). The DCWCO group had lower VISA-A scores at 3 months (56.9 ± 13.9 vs. 65.2 ± 11.0; *P* = 0.044) but higher scores at the latest follow-up (98.2 ± 2.6 vs. 94.3 ± 5.0; *P* = 0.010) than the PPR group. Both groups exhibited significant changes in the Fowler-Philip angle and Bohler’s angle after surgery. The postoperative Fowler-Philip angle of the DCWCO group was greater than that of the PPR group (35.9° ± 4.9° vs. 31.4° ± 6.2°; *P* = 0.026). However, there was no statistically significant difference in any other angle of the two groups postoperatively.

**Conclusions:**

Compared to the PPR group, the DCWCO group had poorer short-term clinical outcomes but provide better long-term function and symptom remission. This method can be a good option for those patients with higher functional expectations.

## Background

Haglund deformity, first described by Patrick Haglund in 1928, is a prominence in the posterolateral heel that causes posterior heel pain [1]. It is generally associated with insertional Achilles tendinopathy (IAT) and retrocalcaneal bursitis, which comprise the Haglund triad or Haglund syndrome [2, 3]. Surgical intervention is a suitable option when conservative treatment for more than 6 months has failed [4]. Common surgical methods, including posterosuperior prominence resection (PPR), retrocalcaneal decompression, and endoscopic treatment, generally have good short-term clinical outcomes [5, 6]. However, a few patients still experience some degree of pain after surgery, especially after PPR [7]; therefore, another surgical method is required for better outcomes.

Dorsal closing wedge calcaneal osteotomy (DCWCO) was first described by Zadek for the treatment of IAT [8]. Keck and Kelly proved that DCWCO was an effective treatment for Haglund deformity in 18 patients [9]. Further, Miller reported that wedge osteotomy of the calcaneus body combined with resection of the superior calcaneus showed good results in 16 patients (18 feet) [10]. Dimitrios treated IAT with dorsal wedge calcaneal osteotomy in 52 athletes, all of whom showed great improvement in both function and pain relief [11]. However, although this surgical technique has been proven to be effective in several studies, a comparison of DCWCO and PPR for the treatment of Haglund syndrome has not been previously reported.

This retrospective review compared the clinical outcomes of DCWCO and PPR with pain relief, improvements in ankle and Achilles tendon function, anatomy changes, and complications as the primary outcomes. Based on these comparisons, we aimed to determine an effective surgical method for treating Haglund syndrome.

## Patients and methods

Patents who suffered the post heel pain during plantar flexion and lift heel for at least 6 months and failed to conservative treatment were suggested to have surgical treatment. They had the option to choose the method after the advantages and disadvantages of both procedures were informed. Records of 50 patients with Haglund syndrome who underwent DCWCO or PPR from January 2009 to October 2014 at the Department of Sports Medicine, Southwest Hospital, Army Medical University (Chongqing, China) were reviewed. Informed consent was obtained from all study subjects. A minimum follow-up of 4 years after surgery was required for all patients.

Specific inclusion and exclusion criteria were determined for selected patients. Inclusion criteria included age older than 18 years and conservative treatment failed. Exclusion criteria included Haglund deformity with Achilles tendon rupture repair, diabetes mellitus with or without neuropathic joint destruction, and local infection. Patients who did not undergo radiography preoperatively or at the 1-year follow-up were excluded. Six patients were excluded due to Haglund deformity with Achilles tendon rupture repair (3 patients), diabetes (1 patient), and incomplete radiography data (2 patients). Forty-four patients were included in this series, and all were divided into the DCWCO group (*n* = 12) or PPR group (*n* = 32). Data regarding age, sex, body mass index (BMI), smoking, operative side, and follow-up duration were collected.

### Operative technique

#### Dorsal closing wedge calcaneal osteotomy

**Fig. 1:**
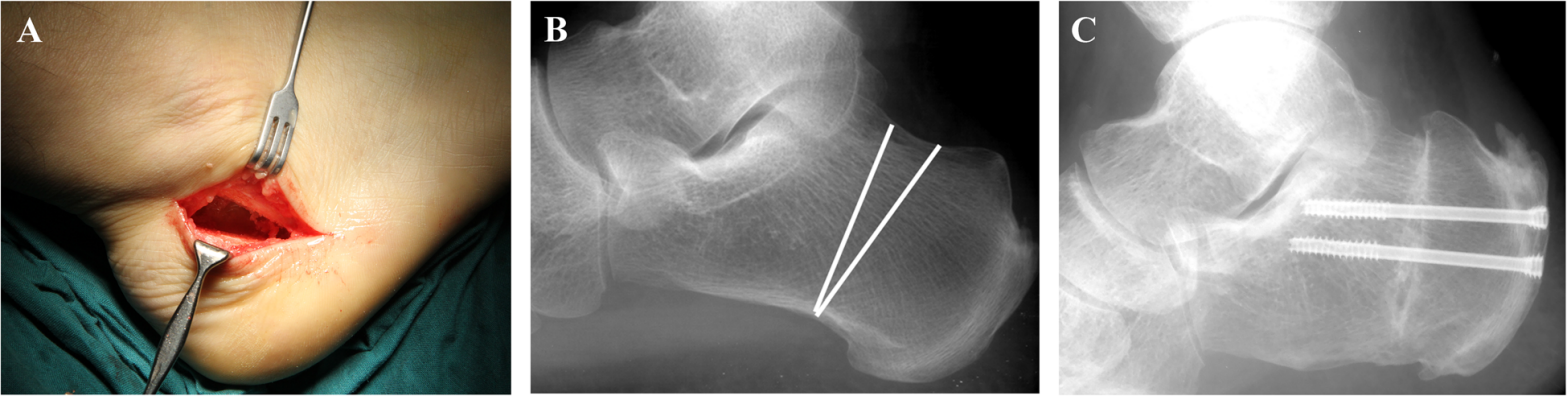
**a** Surgical incision. **b** Cut line of the calcaneus. **c** Two partially threaded cannulated screws used for fixation

**Fig. 2:**
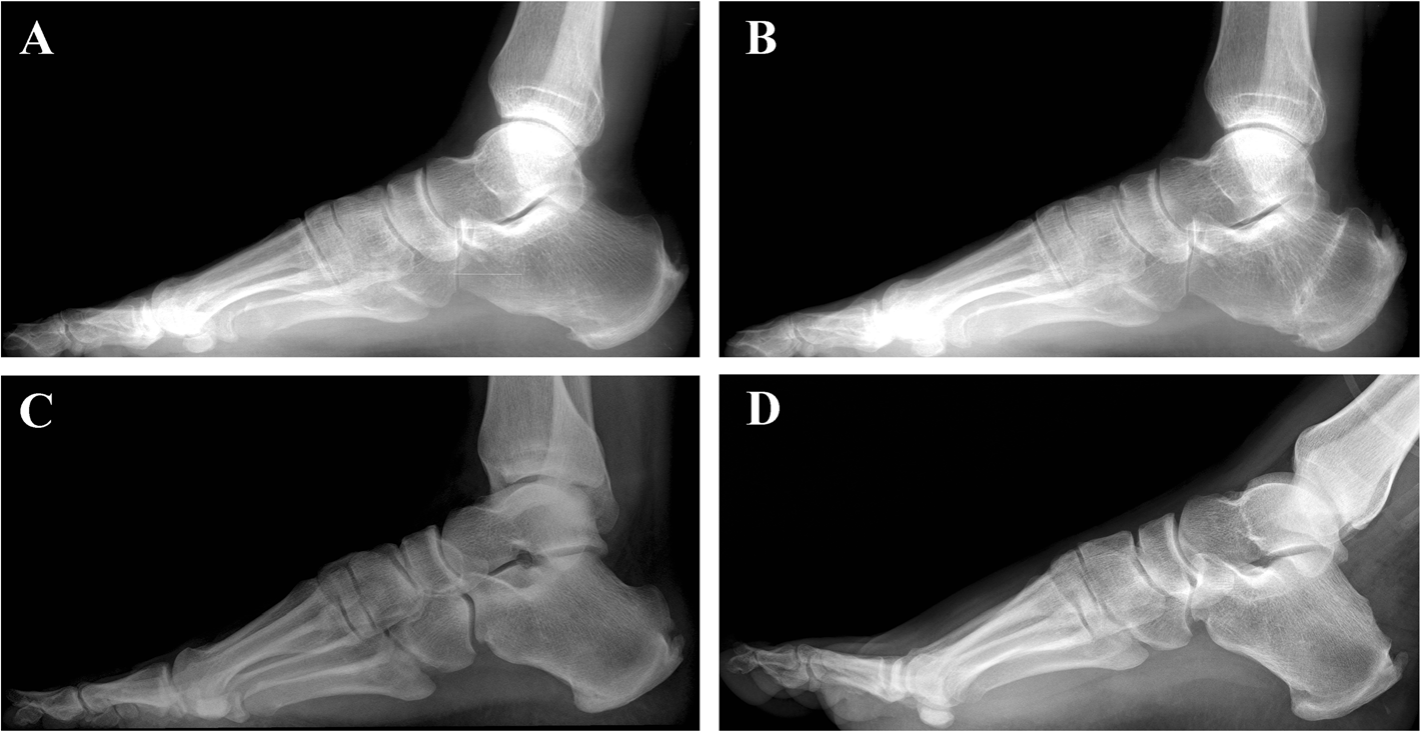
Before and after dorsal closing wedge calcaneal osteotomy (**a** and **b**). Before and after posterosuperior prominence resection (**c** and **d**)

Surgical procedures were performed using a full-thickness lateral approach with patients in the lateral position; regional spinal or epidural anesthesia was administered (Fig. 1a). A thigh tourniquet with 300 mmHg pressure was applied. When the calcaneus was exposed, closing wedge osteotomy was performed for the calcaneal body. The posterior cut was made from a point close to the base of the posterosuperior calcaneal tubercles to the point anterior to the weight-bearing aspect of the plantar calcaneal tubercle. Anterior osteotomy was performed at 90° to the calcaneal underside surface (Fig. 1b). Templates were designed taking into account the angle, orientation, and width of the wedge to be removed according to the preoperative lateral radiograph obtained before surgery. A sagittal saw with a shorter blade was used to complete the osteotomy; then, the calcaneus was fixed with partially threaded cannulated screws under the guidance of two Kirschner wires (Fig. 1c). Final fixation was confirmed using a C-arm device. Then, after closed negative pressure drainage, subcutaneous tissue was repaired with a 3-0 absorbable suture, and the skin was closed with a 3-0 nonabsorbable suture (Fig. 2a, b).

#### Posterosuperior prominence resection

A similar incision but near the tendon insertion was used during PPR with patients in the same position as mentioned previously. After a full-thickness incision was made, the Haglund deformity was clearly exposed. Osteotomy was performed according to the results of preoperative evaluation. The prominence was completely removed distally to proximally using a saw. The Achilles tendon was protected by retractors during the entire procedure. After smoothing the surface, maximal dorsiflexing of the ankle was performed to confirm that there was no obvious impingement between the Achilles tendon and calcaneal surface. Removal of the superior calcaneal prominence was confirmed on C-arm X-ray. After wound irrigation, closed negative pressure drainage was performed. Subcutaneous tissue was repaired with a 3-0 absorbable suture, and the skin was closed with a 3-0 nonabsorbable suture (Fig. 2c, d).

### Postoperative management

For both groups, negative pressure drainage was opened 6 h later after surgery; it was stopped when no active bleeding presented in the drainage. Skin sutures were removed 2 weeks after surgery. In the DCWCO group, weight-bearing was not allowed, and during the initial 4 weeks after surgery, patients were allowed to perform passive motions of the ankle. At 6 weeks to 3 months after surgery, partial weight-bearing with a cast and cane was allowed. After 3 months, patients were allowed full weight-bearing. Patients in the PPR group were told to avoid weight-bearing during the first 3 weeks; however, active range of motion exercises was allowed. After 3 weeks, patients were gradually allowed to walk with the assistance of a cane. Full weight-bearing was allowed at 6 weeks postoperatively.

### Clinical and radiographic evaluations

**Fig. 3:**
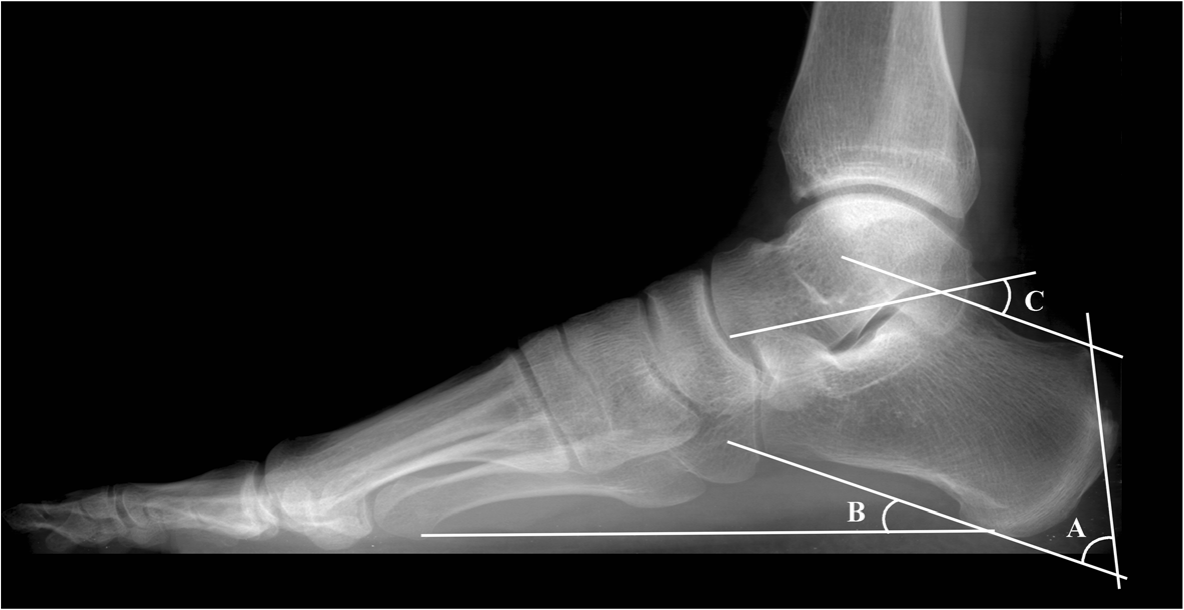
Radiographic indices evaluated using standing lateral foot radiograph. **a** Fowler-Philip angle. **b** Calcaneal pitch angle. **c** Bohler’s angle

Functional evaluations and pain assessments were performed and anatomy changes were observed. Results of the functional evaluations performed preoperatively and postoperatively (at 3 months, 6 months, 1 year, and the latest follow-up) were included to determine the AOFAS score [12] and VISA-A score [13]. Anatomy changes included changes in the Fowler-Philip angle [14], Bohler’s angle [15], and calcaneal pitch angle [5] (Fig. 3). To avoid examiner bias, the clinical evaluation and data analysis were performed by two independent physicians who were blinded to the study.

### Statistical analysis

All data were analyzed statistically using SPSS (version 22.0; IBM, Chicago, IL). Student’s *t* test and a Mann-Whitney’s *U* test were performed to compare the continuous variables. Fisher’s exact test was performed to compare all categorical data. A paired-samples *t* test was used to compare preoperative and postoperative radiologic changes in the same group. Statistical significance was set at *P* < 0.05.

## Results

The series included a total of 44 patients; 12 patients comprised the DCWCO group and the other 32 comprised the PPR group. All patients were normal people, but not athletes, keeping a moderate amount of exercise. Patient demographics, including age, sex, BMI, operative side, smoking, and follow-up time, are summarized in Table 1. There was no significant difference between the two groups in terms of demographic parameters.

**Table 1:** Summary of patient demographics

Demographic variable	DCWCO group (*N* = 12)	PPR group (*N* = 32)	*P* value
Age^a^			0.380
Mean ± SD	32.8 ± 9.7	36.5 ± 14.1	
Median	36.5	36.5	
Range (minimum-maximum)	18–44	18–63	
Gender^b^			0.647
Female, *n* (%)	3 (25.0)	6 (23.1)	
Male, *n* (%)	9 (75.0)	26 (76.9)	
BMI^a^			0.399
Mean ± SD	24.4 ± 2.8	23.5 ± 3.1	
Median	24.1	23.8	
Range (minimum-maximum)	19.03–28.73	18.37–31.88	
Operative side^b^			0.504
Right	5 (41.7)	18 (56.3)	
Left	7 (58.3)	14 (43.7)	
Smoking^b^			0.579
Smoker	3 (25.0)	9 (28.1)	
Non-smoker	9 (75.0)	23 (71.9)	
Follow-up duration (months)^a^			0.055
Mean ± SD	86.5 ± 17.1	71.8 ± 22.4	
Median	81.5	76.5	
Range (minimum-maximum)	65–116	40–120	

^a^Student *t* test and ^b^Fisher’s exact test were used to compare both groups and no statistically significant differences were observed (*P* > 0.05)

**Fig. 4:**
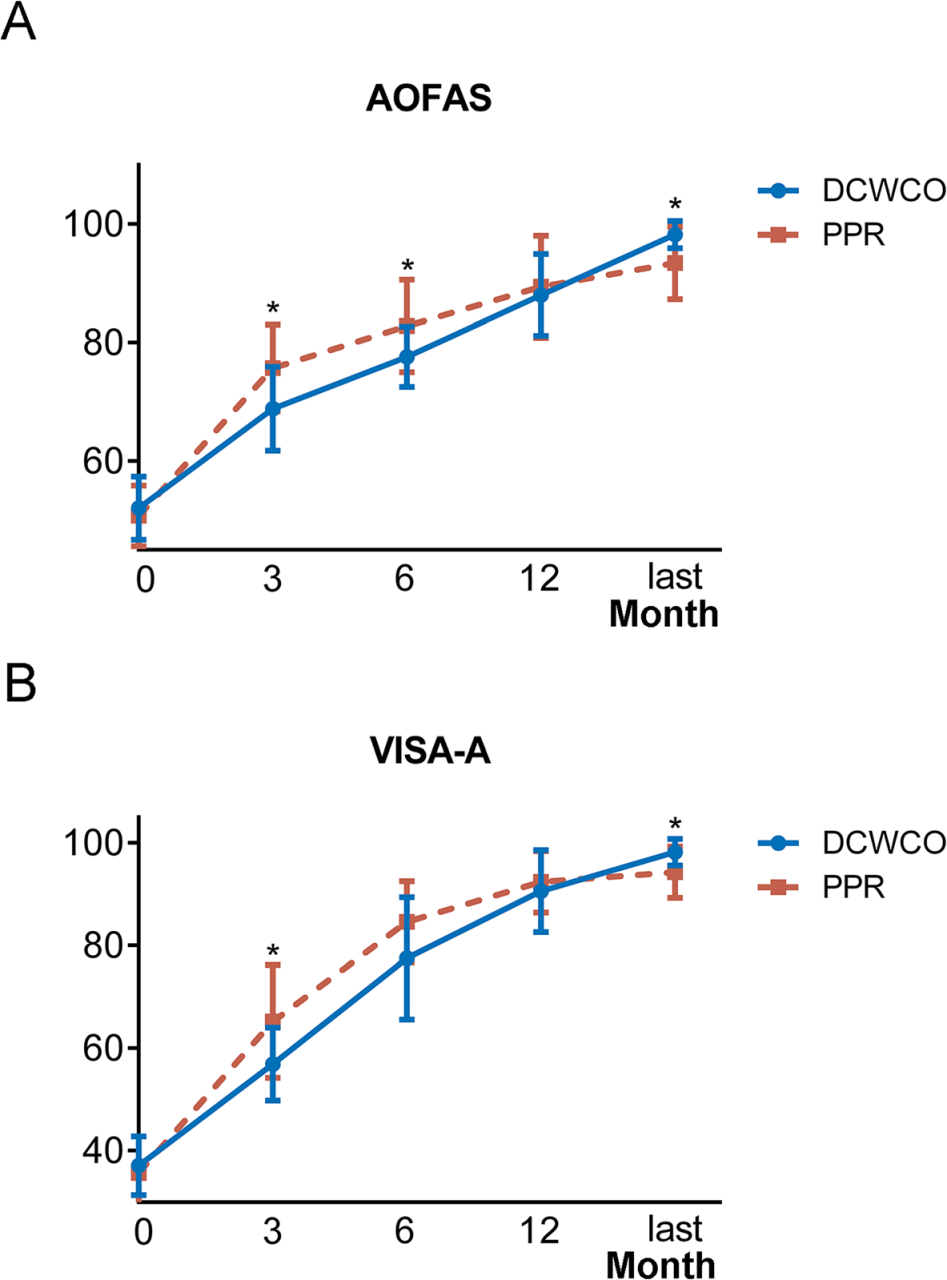
The comparison outcomes of two groups at preoperatively, 3, 6, 12 months, and last follow-up. **a** AOFAS score. **b** VISA-A score. ^*^Significant difference between the two groups

All preoperative and postoperative (at 3 months, 6 months, 1 year, and the latest follow-up) function scores for both groups are summarized in Table 2. The mean AOFAS and VISA-A scores were not significantly different between the two groups preoperatively. During an average follow-up of 86.5 months, the AOFAS score of the DCWCO group increased from 52.0 ± 5.3 preoperatively to 98.2 ± 2.3 at the latest follow-up. The mean AOFAS score of the PPR group increased from 50.7 ± 5.1 preoperatively to 93.4 ± 6.1 at the latest visit during an average follow-up of 71.8 months. A comparison of the AOFAS scores of the two groups at 3 months and 6 months postoperatively showed that the scores of the DCWCO group were significantly lower than those of the PPR group. However, the scores of the DCWCO group at the latest follow-up were significantly better than those of the PPR group. A similar trend was also seen in the VISA-A scores; scores were only different at the 6-month follow-up. The PPR group had a mean score of 84.6 (± 7.9), while that of the DCWCO group was 77.5 (± 11.9); although the score of the PPR group was better, it was not statistically different. (Fig. 4)

**Table 2:** Comparison of functional scores pre- and postoperatively for both groups

Scale	DCWCO group (*N* = 12)	PPR group (*N* = 32)	*P* value
AOFAS score			
Preoperatively^a^	52.0 ± 5.3	50.7 ± 5.1	0.464
3 months^a^	68.8 ± 7.1	75.7 ± 7.3	0.007^c^
6 months^a^	77.6 ± 5.1	82.8 ± 7.8	0.037^c^
1 year^a^	88.0 ± 6.9	89.4 ± 8.6	0.607
Latest follow-up^b^	98.2 ± 2.3	93.4 ± 6.1	0.030^c^
VISA-A			
Preoperatively^a^	37.1 ± 5.7	35.7 ± 7.1	0.530
3 months^a^	56.9 ± 13.9	65.2 ± 11.0	0.044^c^
6 months^b^	77.5 ± 11.9	84.6 ± 7.9	0.118
1 year^a^	90.6 ± 8.0	92.4 ± 6.0	0.427
Latest follow-up^b^	98.2 ± 2.6	94.3 ± 5.0	0.010^c^

^a^Student *t* test. ^b^Mann-Whitney *U* test. ^c^Significant difference between the two groups

The preoperative and postoperative radiologic parameters of both groups, including the Fowler-Philip angle, Bohler’s angle, and calcaneal pitch angle, are summarized in Table 3. The Fowler-Philip angle significantly decreased and Bohler’s angle significantly increased in both groups after surgery; however, the calcaneal pitch angle did not change.

**Table 3:** Comparison of the radiologic indices pre- and postoperatively for both groups

Measure	DCWCO group (*N* = 12)	PPR group (*N* = 32)	*P* value
Fowler-Philip angle			
Preoperatively^a^	54.0° ± 5.2°	53.0° ± 5.6°	0.628
Postoperatively^a^	35.9° ± 4.9°	31.4° ± 6.2°	0.026^c^
Bohler’s angle			
Preoperatively^a^	32.1° ± 3.3°	32.0° ± 3.4°	0.994
Postoperatively^b^	43.6° ± 2.8°	44.0° ± 4.7°	0.726
Calcaneal pitch angle			
Preoperatively^a^	25.5° ± 1.9°	25.1° ± 3.3°	0.704
Postoperatively^a^	25.4° ± 1.8°	24.9° ± 2.4°	0.587

^a^Student *t* test. ^b^Mann-Whitney *U* test. ^c^Significant difference between the two groups

The postoperative Fowler-Philip angles were significantly larger in the DCWCO group than in the PPR group. All other angles of the DCWCO group were not statistically different when compared to those of the PPR groups preoperatively and postoperatively.

Postoperative complications developed in one patient in the DCWCO group and one patient in the PPR group. One patient in the DCWCO group experienced delayed union of the calcaneus until 4 months after surgery; however, 1 year later, after the screws were removed, his pain was completely relieved. One patent in the PPR group developed a *Staphylococcus aureus* infection at the incision site; however, the incision healed well after infusion treatment with cefazolin five days, and no further debridement was performed.

## Discussion

The most important finding of this study was that the DCWCO group had poorer short-term clinical outcomes but better long-term function and symptom remission than the PPR group after treatment for Haglund syndrome. Both surgical methods decreased the Fowler-Philip angle and resulted in no statistical differences in other angles.

According to the results, both groups showed significant pain relief and function improvement, and few surgical complications were noted. The PPR group had better outcomes than the DCWCO group during short-term follow-up. Patients in the PPR group were nearly pain-free after surgery and able to quickly return to their previous sports activities. The DCWCO group, however, required a longer period of recovery because of the more severe trauma caused by the surgery. However, most patients in the DCWCO group experienced pain relief by 1 year postoperatively. The majority of patients experienced complete recovery and were able to perform exercise without any concerns by the time of the last follow-up. In general, the DCWCO group had poorer short-term outcomes but better long-term clinical outcomes when compared with the PPR group.

Haglund deformity is an abnormality of the posterosuperior part of the calcaneus [16]. Because it is near the insertion of the Achilles tendon, the adjoining tendon and soft tissue are irritated and compressed during ankle motion [17]. This impingement is considered the primary cause of Achilles tendinopathy [4, 18]. Surgical treatment to remove the impingement is required when altering the heel height, physiotherapy, local steroid injections, and other conservative treatments fail. PPR can effectively eliminate the impingement between the Haglund deformity and Achilles tendon. However, the impingement between the insertion of the Achilles tendon and calcaneus can persist regardless of the extent of resection. Therefore, changing the structure of the calcaneus may be another treatment option. The Fowler-Philip angle was reduced by DCWCO, and the direction of the prominence was also altered. Therefore, with DCWCO, there was no need for additional resection of the Haglund deformity. Further, both areas of impingement were avoided during the motion of the Achilles tendon. Additionally, the insertion of the Achilles tendon was slightly elevated due to the osteotomy, and the orientation of the tendon at the insertion was changed. These biomechanical alterations may reduce tendon stress [19] and help eliminate risk factors and modulate the progress of Achilles tendinopathy. However, an effective and rational biomechanical design is required for successful anatomical changes in tendon insertion.

The different management methods used for the soft tissue near the tendon could explain the results of this study. Compared with DCWCO, PPR can cause injury to the soft tissue. Furthermore, Kager’s fat pads are believed to be associated with Achilles tendinopathy, and the size of the fat pads differs between individuals with and without tendinopathy [20]. Cytokines produced by Hoffa’s fat pad have a vital role in knee osteoarthritis [21], and the potential role of fat pads in mediating angiogenesis may aggravate the progression of Achilles tendinopathy [22]. Therefore, PPR may affect the recovery in Achilles tendinopathy by injuring the fat pad near the tendon, thus contributing to worse long-term clinical outcomes.

Different osteotomy methods and recommendations for performing DCWCO have been reported. Boffeli set the anterior osteotomy at 90° to the weight-bearing surface at the plantar apex and made the posterior cut from the calcaneal tubercles to the point posterior to the weight-bearing aspect of the plantar calcaneal tubercle [23]. This approach had a slight impact on the insertion of the plantar fascia and the anatomic shape of plantar tubercles. However, more attention should be focused on protecting the Achilles tendon during this procedure. Additionally, less bone fixation necessitates less obtrusive surgery. Georgiannos et al. performed a similar osteotomy as we did; they used K-wire to persevere the plantar bone-hinge [24] and showed good clinical outcomes in 64 feet of athletes [11]. Maffulli et al. coupled a resection of the posterosuperior corner of the calcaneus with a dorsally based closing wedge osteotomy of the calcaneus, placed more posterior to the one we undertook, without debriding the insertion of the Achilles tendon, with significant functional improvement at 2 years after the procedure [25]. Their methods and ours are both options to perform this surgery with less complications and more reproducibility.

Upon review of all patients who underwent surgery for Haglund syndrome, no patients who underwent insertional Achilles tendon rupture repair with an anchor were noted in the DCWCO group due to the limitation of the incision. Furthermore, the outcomes of patients who underwent deformity resection and anchor reconstruction were generally worse than the outcomes of those who underwent only deformity resection. We believe that this difference may be attributable to the ruptured insertional tendon. Therefore, we excluded patients who underwent tendon rupture repair to avoid bias and interference. What is more, patients with severe calcified insertional Achilles tendinopathy had been evaluated before surgery. These cases requiring debridement were suggested to undertake PPR due to the limitation of DCWCO incision. However, patients with mild symptom of calcified insertional Achilles tendinopathy would still be suggested of two methods. When these patients undertook DCWCO, the calcific deposits would not be removed. The outcomes of these patients showed excellent in the long-term follow-up. It indicated that the progress of calcific insertional Achilles tendinopathy can be slow and even reverse when calcaneal anatomy changed. However, this conjecture needs further study to prove. Although the incision we chose for DCWCO was not suitable for repairing the tendon with an anchor, we believe that a combination of DCWCO and tendon repair to treat Haglund deformity with tendon rupture may be feasible. Maffulli et al. have reported a transverse incision in treatment of IAT. This incision provided a bordered surgical field, making an adequate debridement and less soft tissue injury to be possible [26]. This incision probably can be combined with DCWCO; however, the surgery technique and long-term outcomes will need to be established and proved through further studies.

This study has some limitations. First, in DCWCO, insertion of the Achilles tendon was mildly increased due to osteotomy; however, the effects of an anatomy change have not been proven by biomechanical testing. Further, although no symptoms were observed during long-term follow-up after DCWCO in this study, they cannot be ignored in actual clinical practice. The good clinical outcomes may be attributable to adaptation of the tendon to structural changes and release of tendon stress following DCWCO. And the outcomes can be more convincible if a culturally validated version of assessment scoring is used. Further, the small sample size and long-term follow-up may also be factors responsible for the good clinical outcomes noted in this study. Therefore, an effective and rational biomechanical design is required for future application of this procedure. Furthermore, although the calcaneal union did not occur in our patients, it is still a potential surgical complication that remains a challenge for surgeons, especially for those performing this surgery for the first time. Additionally, early functional rehabilitation was required with DCWCO due to the longer recovery period, and the second surgery for removal of the two screws increased the associated cost and pain.

## Conclusion

This study compared DCWCO and PPR for the treatment of Haglund syndrome. DCWCO resulted in poorer short-term clinical outcomes but better functional improvement than PPR during long-term follow-up. This method can be a good option for those patients with higher functional expectations.

## Data Availability

All data and materials regarding the study are available from the corresponding author.

## Abbreviations

DCWCO: Dorsal closing wedge calcaneal osteotomy
PPR: Posterosuperior prominence resection
IAT: Insertional Achilles tendinopathy
AOFAS: American Orthopedic Foot Ankle Society ankle-hindfoot scale
VISA-A: Victorian Institute of Sport Assessment Scale for Achilles tendinopathy
